# Spontaneous motor tempo over the course of a week: the role of the time of the day, chronotype, and arousal

**DOI:** 10.1007/s00426-022-01646-2

**Published:** 2022-02-06

**Authors:** David Hammerschmidt, Clemens Wöllner

**Affiliations:** Institute for Systematic Musicology, University of Hamburg, Alsterterrasse 1, 20354 Hamburg, Germany

## Abstract

The spontaneous motor tempo (SMT) or internal tempo describes the natural pace of predictive and emergent movements such as walking or hand clapping. One of the main research interests in the study of the spontaneous motor tempo relates to factors affecting its pace. Previous studies suggest an influence of the circadian rhythm (i.e., 24-h cycle of the biological clock), physiological arousal changes, and potentially also musical experience. This study aimed at investigating these effects in participants‘ everyday life by measuring their SMT four times a day over seven consecutive days, using an experience sampling method. The pace of the SMT was assessed with a finger-tapping paradigm in a self-developed web application. Measured as the inter-tap interval, the overall mean SMT was 650 ms (*SD* = 253 ms). Using multi-level modelling (MLM), results show that the pace of the SMT sped up over the course of the day, and that this effect depended on the participants’ chronotype, since participants tending towards morning type were faster in the morning compared to participants tending towards evening type. During the day, the pace of the SMT of morning types stayed relatively constant, whereas it became faster for evening-type participants. Furthermore, higher arousal in participants led to a faster pace of the SMT. Musical sophistication did not influence the SMT. These results indicate that the circadian rhythm influences the internal tempo, since the pace of SMT is not only dependent on the time of the day, but also on the individual entrainment to the 24-h cycle (chronotype).

**Supplementary Information:**

The online version contains supplementary material available at 10.1007/s00426-022-01646-2.

## Introduction

The tempo of spontaneous motoric movements plays an important role in everyday life as it represents the unconsciously chosen pace for regular and repeated movements such as walking or hand clapping. In the study of spontaneous motor tempo (SMT), one of the main research questions relates to factors influencing its pace. The SMT typically clusters around 2 Hz (Collyer et al., 1994; Fraisse, 1982), yet intra-individual changes in the pace of the SMT have been suggested to be based on the circadian rhythm (Hammerschmidt et al., 2021; Moussay et al., 2002). The purpose of the current study was to further investigate this effect by measuring participants’ SMT four times a day over seven consecutive days in their everyday life. Furthermore, since there are different chronotypes, a potential influence of participants’ individual 24-h sleep and activity cycle (i.e., chronotype) was also investigated. As previous studies did find differences in the pace of the SMT based on physiological arousal changes (Boltz, 1994; Dosseville et al., 2002), and musical experience (Drake et al., 2000; Hammerschmidt et al., 2021), participants’ arousal level and musical sophistication were also taken into account as further predictors.

The SMT corresponds to the preferred and natural pace of periodic motor actions (i.e., cyclic movements), and therefore it is also called preferred or internal tempo (Boltz, 1994). This preference can be observed in synchronization-continuation motor tasks, for instance. When synchronizing to external events consisting of a steady pulse at rates faster or slower than the SMT, individuals eventually fall back into the pace of their internal tempo in the continuation phase (McAuley et al., 2006; Yu et al., 2003). The SMT is closely linked to the preferred tempo for the perception of rhythmical structures in music and language, for which temporal discrimination abilities are optimal, suggesting a shared mechanism for perceptual and rhythmic motor behaviors (i.e., *Preferred Period Hypothesis;* McAuley et al., 2006; McAuley & Jones, 2003; Michaelis et al., 2014). Furthermore, the pace of the SMT may reflect the rate of internal time and timing mechanisms facilitating attentional synchrony (Jones & Boltz, 1989; McAuley & Jones, 2003). Hence, the SMT can be seen as an estimate of an intrinsic timekeeper (Allman et al., 2014; Boltz, 1994; Grondin, 2010; Treisman, 1963). Although there is a large variability in the pace of the SMT ranging from 190 to over 1000 ms, the optimal tempo of rhythm perception, the execution of predictive (rhythmic), and emergent (cyclic) movements such as finger tapping, stepping, or walking all have been shown to share the same resonance frequency at around 2 Hz (Collyer et al., 1994; Goodman et al., 2000; Rose et al., 2021; van Noorden & Moelants, 1999). This resonance frequency may be under the control of the central nervous system reflecting the most stable state for movement trajectories (Assaneo & Poeppel, 2018).

Changes in the SMT indicate that its pace is not fixed at a certain tempo but subject to intra-individual changes. One of the potential factors causing these changes is the circadian rhythm. This 24-h cycle of the low-level biological clock has been shown to affect multiple cognitive and physiological functions such as motor processes, reaction times, time judgments, and memory tasks (Valdez et al., 2012). Investigating the circadian fluctuation of the SMT, Moussay et al. (2002) measured SMT in cycling and finger tapping five times a day between 6 am and 10 pm showing that the pace of SMT got faster during the day (6 am to 6 pm) and slowed down during the evening (6 to 10 pm). This result suggests a direct influence of the circadian rhythm on the SMT. Further support for this stems from a large-scale online study with *N* = 3756 participants investigating effects of psychological, chronobiological, and demographic factors on the SMT (Hammerschmidt et al., 2021). In this study, participants tapped at a tempo that felt most natural and comfortable in that moment on a device of their choice using a browser-based web application. Participants were grouped into six clusters which differed in terms of their SMT pace ranging from very fast (*M* = 265 ms, *SD* = 74) to very slow (*M* = 1757 ms, *SD* = 166). Results show that the slower the SMT cluster, the earlier was the mean hour of test participation, thus further supporting a direct influence of the biological clock on the SMT pace. Furthermore, a recent study found that musicians’ spontaneous production rate (i.e., tempo) of musical melodies varies as a function of the time of the day, as melody production was slower in the morning (9 am) compared to later times (1 pm, 5 pm, 9 pm; Wright & Palmer, 2020). Another study on the cognitive output close to sleep phases investigated the tapping speed of smartphone usage for about three weeks (Huber & Ghosh, 2021). Although not directly comparable to predictive and emergent movements, results show that finger tapping speed on the smartphone (i.e., typing) got faster during the morning hours, remained relatively constant during the day, and slowed down during the night. These studies show that the pace of the SMT as well as general finger movements vary during the day, and that these changes may be caused by the circadian rhythm.

An important aspect which has not been addressed in previous research on the relationship between the SMT and the circadian rhythm is the role of inter-individual differences in the internal clock and its entrainment to circadian rhythm. Individual preferences in the sleep and activity cycle are commonly referred to as chronotypes. These types differ in terms of the phase reference, meaning the midpoint between sleep onsets, which is shifted between them (Roenneberg et al., 2003). Chronotypes are often described on a spectrum between morning types or “larks” (early sleepers) and evening types or “owls” (late sleepers). The chronotype may further explain differences in the fluctuations of the SMT over the course of a day, because clock times for the fastest and slowest SMT may be shifted between morning and evening types. Support for such chronotype-induced differences stems from a study on piano playing. Although not directly investigating the pace of the SMT, the timing (variability) of evening-type pianists was reported to be more stable when playing in the evening than in the morning (van Vugt et al., 2013). Thus, timing mechanisms at different times during the day may be affected by the chronotype. Furthermore, other chronotype-dependent performance differences have been shown to exists in memory (Intons-Peterson et al., 1999; West et al., 2002), attention (Matchock & Mordkoff, 2009), and sensorimotor tasks (Tamm et al., 2009). Thus, it seems reasonable to assume an influence of the chronotype on the circadian fluctuation of the SMT.

Another important intra-individual factor which may affect the SMT is arousal. According to the *Sympathetic Hypothesis*, higher physiological arousal increases the preferred perceptual tempo (Jakubowski et al., 2015) and may in turn affect the SMT as well (Holbrook & Anand, 1990). In line with this, studies found that varying induced arousal levels from auditory stimuli affected both the SMT and duration judgments (Boltz, 1994). High arousal stimuli led to a faster SMT and shorter duration judgments compared to low arousal stimuli. On the other hand, in another study on the effects of perceived arousal in music and cognitive load, no differences in duration judgments were reported between high and low arousal music when the tempo was kept constant, and no influence of the SMT was found in contrast to significant effects of attention and cognitive load on time perception (Wöllner & Hammerschmidt, 2021). Regarding felt arousal in contrast to induced arousal, an online-study measuring SMT using a finger-tapping paradigm showed that participants who rated their arousal level as high showed a faster SMT as well (Hammerschmidt et al., 2021). Physiological changes in the body (e.g., heart rate, cortical blood flow) evoked by physical activity are closely related to the arousal level (Fisher, 2014; Nobrega et al., 2014), yet the relationship between physiological arousal and SMT remain inconclusive. Whereas one study did find a faster SMT after a pedaling exercise (high physiological arousal) compared to before the exercise (Dosseville et al., 2002), no such faster pace was reported in a study in which participants performed either swimming, running, or wrestling tasks (Sysoeva et al., 2013). Furthermore, the effect of arousal may also be affected by the chronotype, as it has been shown that morning-type individuals have a higher skin conductance (i.e., physiological arousal) in the morning than evening types and vice versa (Wilson, 1990). Thus, further research is needed on the effect of arousal on the pace of the SMT, and potential changes based on the chronotype.

Apart from effects with regard to the biological clock (i.e., circadian rhythm, chronotype), other inter-individual factors have been shown in relation to the pace of the SMT. Perhaps the most consistently reported effect influencing the SMT is age. Multiple studies showed that the pace of the SMT slows down with higher age (Baudouin et al., 2004; Hammerschmidt et al., 2021; McAuley et al., 2006), either reflecting a slowing down of internal timing processes or a decline in cognitive and behavioral speed caused by changes in the neuromuscular system (Salthouse, 2010; Seidler et al., 2010; Surwillo, 1968). Musical experience has also been suggested to have an effect on the SMT. Children with musical training showed a slower SMT compared to children without such training in a study on the development of rhythmic attending (Drake et al., 2000). However, this difference dissolved for adults, whereas another study did find differences in the pace of SMT in adults based on musical experience (Hammerschmidt et al., 2021). In this study, participants with a slow SMT had the least musical experience. Yet, results regarding a potential influence of musical experience remained inconclusive, as more musically experienced participants generally preferred a slower SMT across all SMT clusters. Furthermore, a potential effect of musical experience lacks a detailed explanation. In contrast, for tasks involving sensorimotor coupling such as synchronization with an auditory beat or music benefit from musical training and enhance the ability to track auditory-motor events over a longer time span (Hammerschmidt & Wöllner, 2020; Repp, 2010; Scheurich et al., 2018). It can be assumed that SMT reflects the pacing of a rather low-level biological clock that should not rely on higher-level cognitive capabilities such as in sensorimotor synchronization (SMS) in young and middle-aged healthy adults. Thus, more research is needed in order to validate if musical experience slows down the pace of the SMT and reduces variability.

To sum up, previous research suggests a potential influence of the circadian rhythm on the SMT, since the pace of the SMT may fluctuate as a function of the time of the day (Hammerschmidt et al., 2021; Moussay et al., 2002). As different chronotypes show a shift in the biological clock and its entrainment to the sleep and activity cycle (Roenneberg et al., 2003), it seems likely to assume that individual differences in the circadian fluctuation depend on the chronotype as well. In addition, higher arousal levels have been shown to cause the SMT to get faster in pace, yet studies resulted in diverging results for physiological activation, induced and perceived arousal (Boltz, 1994; Dosseville et al., 2002; Sysoeva et al., 2013; Wöllner & Hammerschmidt, 2021). Furthermore, musical experience may have an effect on the SMT (Drake et al., 2000; Hammerschmidt et al., 2021).

The current study aimed at investigating whether the SMT is systematically influenced by time of the day; a potential interaction with the chronotype, as well as the arousal level. Furthermore, participants’ musical sophistication as a measure of experience was collected in order to control for potential differences in the SMT. We hypothesized that (i) the time of the day influences the pace of the SMT, (ii) the individual chronotype changes the effect of the time of the day on the pace of the SMT by shifting its phase (interaction effect), and that (iii) higher arousal leads to a faster SMT. These factors were measured in participants’ everyday life and thus in an out-of-the-lab context using an experience sampling method. SMT was assessed with a finger-tapping paradigm, by letting participants tap on the touchscreen of their smartphone using a self-developed web application. Furthermore, an analysis of the same factors on tapping variability (coefficient of variation) was carried out.

## Method

### Participants

A total of 36 participants (21 female) took part in the study. Participants’ age was between 19 and 40 years (*M* = 28.64, *SD* = 5.00). Eight participants were university students or on job training, four participants were unemployed, and the others were either full or part time working (66.7%). All participants were German residents. Their average musical sophistication was *M* = 71.11 (*SD* = 23.91, range: 26–115), which is slightly below the average of the general population (*M* = 81.58, *SD* = 20.62) as measured with the general factor of the *Musical Sophistication Index* (Gold-MSI; Müllensiefen et al., 2014). The average D-MEQ score as a measure of participants’ chronotype was *M* = 48.44 (*SD* = 10.52), showing no bias towards a particular chronotype in the whole sample (see supplementary materials). Participants were recruited from a larger pool of individuals who took part in an online pre-study using the same finger-tapping paradigm (*N* = 49). Criteria for exclusion from the main study were as follows: (1) large variability in their finger tapping assuming false task execution, (2) age (> 40 years), as previous studies showed a slowing with age (e.g., McAuley et al., 2006), and (3) being on vacation during the test period, as vacation might change the typical sleep and activity cycle compared to a typical week. All participants gave informed consent online and procedures were in accordance with the guidelines of the Ethics Committee of the Faculty of Humanities at University of Hamburg. Participants were compensated with 30€.

### Design and procedure

In this study, an experience sampling method was used by prompting participants on their smartphone via text message four times a day over seven consecutive days (Monday–Sunday). This resulted in 28 repeated measures per participant. Before the start of the test period, participants were asked to fill out a questionnaire consisting of multiple inventories (see supplementary materials). Due to the general situation regarding the COVID-19 pandemic in Germany at the time of the experiment (20th–26th of July, 2020), all study procedures (declaration of participation, general introduction, example test) were carried out virtually.

Following the invitation to take part in the study, participants gave informed consent and filled out an online questionnaire using SoSci Survey. In this questionnaire, participants entered information on their age, gender, education, and current occupation. They also filled out the German version of the *Morning-Evening Questionnaire* (D-MEQ) in order to assess participants’ chronotype (Griefahn et al., 2001). They also answered the questions from the general factor of the *Musical Sophistication Index* (Müllensiefen et al., 2014). In the next step, all participants took part in one of two general introduction events via a video conference tool in which they were informed about the test procedure during the week and the main finger-tapping task. This introduction took part on the Thursday and Friday before the start of the test week on the following Monday. During the weekend, they received a first text message including the link to the study in order to familiarize them with the test procedure and tasks. Starting on the Monday, participants received a text message on their smartphone prompting them to do the test as soon as possible by clicking on the attached link. These text messages were randomly sent between 8 and 9 am, 12 and 1 pm, 4 and 5 pm, as well as 8 and 9 pm to all participants, in order to prevent them from always responding at exactly the same time and anticipating the task.

In each test, participants’ SMT was measured first by asking them to tap evenly for 25 s with their index finger of their preferred hand on the touchscreen of the smartphone at a pace that felt most comfortable and natural to them in that moment. If the tapping was too irregular (coefficient of variation of inter-tap intervals > 30%) or not enough taps were recorded (< 8 taps), they were asked to repeat the task. After the tapping task, participants answered questions about their current activity, location, and company (see supplementary materials). They filled out the 7-item *Physiological Arousal Questionnaire* (PAQ) using a 7-point scale instead of the original 9-point scale (Dieleman et al., 2010). This adjustment was needed in order to fit the graphical representation of the rating scale on smartphone displays. On average, participants needed 1.07 trials (*SD* = 0.33) to reach criteria for a successful tapping task execution. The browser-based web application used for the recording of the finger taps and the other responses was the same as in Hammerschmidt et al. (2021), apart from changes regarding additional questions to account for the different scope of this study. The script for the application is publicly available and can be accessed on Github (https://github.com/g-mac/slomo).

### Data processing

Participants’ SMT was assessed by taking the mean of the inter-tap intervals (ITI) for each test in milliseconds. Before statistical testing, single cases were excluded when a test was incomplete or missed by a participant (*N* = 62). Next, separate outlier detections for each participant on their ITIs were performed using 1.5 interquartile range, resulting in *N* = 17 further cases being excluded. From the 1008 tests (36 × 28), 6.2% were missing or excluded, resulting in an average response rate of 93.8% per participant. Out of the 28 tests per participant, the minimum number of tests was 24 (85.7%). The scores for participants’ chronotype (D-MEQ score), arousal (PAQ score), and general musical sophistication (general factor Gold-MSI score) were calculated according to the respective specifications. The actual times of test execution were converted to the relative times from the first text prompt from each day by subtracting the time of the first text message (between 8 and 9 am) from the times of the four tests per day. These time differences were then converted to decimals thus, a test at 10:30 pm and 1:30 h after a first prompt equals 1.5. This conversion allows for the assessment of the actual time of participants’ responses instead of grouping them, controlling for differences in response times and allowing for the treatment of the time of the day as a continuous variable (cf., Smith et al., 2019). This procedure resulted in the exclusion of one more case since the corresponding test was done before the first prompt of the day (indicated by a negative time of the day value). In a last step, fixed variables were centered before performing multi-level modelling: the within-participants (level-1) variable PAQ score (assessed with each test) was centered-within-clusters (cwc approach) and between-participant variables D-MEQ and Gold-MSI (level-2, assessed before the test period) were grand-mean-centered (gmc approach) since 0 is not a meaningful score in these inventories. Thus, the SMT intercept of the reported multi-level model represents the average SMT between 8 and 9 pm when arousal score (PAQ) equals the participant’s average arousal across the week as well as the mean chronotype score (D-MEQ) and musical sophistication score (Gold-MSI) across all participants (Curran & Bauer, 2011; Nezlek, 2012). In the end, analysis was based on *N* = 946 test cases. Data processing, model building, and statistical testing were done in R (R Core Team, 2020).

### Model building

**Fig. 1:**
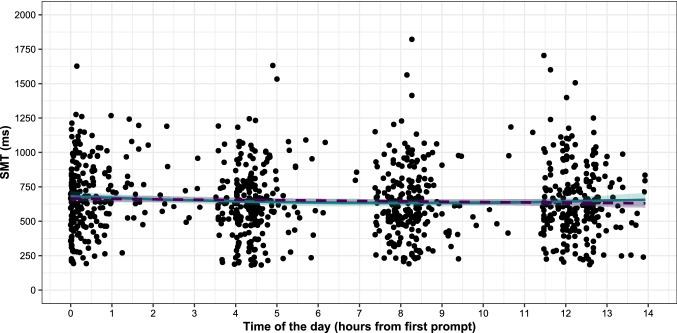
Participants’ SMT in milliseconds plotted against the time of participation after the first prompt between 8 and 9 am (value 0) for all seven days. The lines represent regression lines (dashed purple = linear, solid green = polynomial)

In order to assess the effects of time of the day, arousal (PAQ cwc-score), chronotype (D-MEQ gmc-score), and musical sophistication (Gold-MSI gmc-score) on the pace of the SMT, a multi-level model was applied regarding the mean ITI as a dependent variable using the *lme4* package in R. This statistical test was chosen to account for the hierarchical structure of experience sampling data (i.e., each participant provided up to 28 responses). In order to test our hypotheses and to find other potential interaction effects, a model building process was applied which resulted in four different models differing in the number of parameters (see Table 1). This approach allowed for an informed data-driven choice regarding which interactions to include in the final model and offers a broader model performance context. In a first step, it was checked if the pace of the SMT differed between days, which was not the case, *β*  = − 2.20, *t*(910.21) = 0.86, *p* = 0.39, and, therefore, the days were not included in the models. Next, an unconditional model with participants as random factor was performed as a baseline model and the intraclass correlation coefficient (ICC) was calculated. Then, the level-1 variables time of the day and arousal were added as random effects. The random effects covariance matrices resulted in singular fits (i.e., random variance close to 0) and were, therefore, not further included as random factors in the model building process. In the next step, a model with all variables (level-1: time of the day, arousal; level-2: chronotype, musical sophistication) as fixed factors was calculated. In order to check for potential interaction effects between the variables, all possible interactions were included in the model. In order to avoid overfitting of the model, only two-way interactions were considered. All non-significant interactions (*p* > 0.05) were then removed again from the model, as it significantly improved model prediction and in order to achieve a more parsimonious model. In a last step, it was checked if the time of the day variable should be included as a polynomial term as well, which was not the case as this time factor was not significant, *β* = 0.47, *t*(910.43) = 1.51, *p* = 0.13 (see Fig. 1), and this did also not significantly improve model performance (*p* = 0.16; see Fig. 1). Therefore, time of the day was treated as a linear variable.

**Table 1:** Performance measures for the multi-level model building

Models	Number of parameters	AIC	BIC	Log Likelihood	Deviance	*p*
Unconditional	3	12,379	12,393	− 6186	12,373	
Fixed factors	7	12,368	12,402	− 6177	12,354	0.001
Fixed factors + sig. interactions*	8	12,366	12,405	− 6175	12,350	0.044
Fixed factors + all interactions	13	12,373	12,436	− 6174	12,347	0.716

Model comparisons (*p*-values) were done sequentially to the one below. The asterisk indicates the best performing and final model

Table 1 shows the performance measures and sequential comparisons (significance tests) of the four models sorted by the number of parameters. The best performing model of that list was then chosen for reports of parameter predictions and to validate hypotheses. If an interaction was non-significant (*p* > 0.05), it was not included in the model. The final model resulted in the following equations:

﻿Level-1:﻿

1 $$y({\mathrm{SMT})}_{ij}={\beta }_{0j}+{\beta }_{1j}{(\mathrm{Time\,of\,the\,day})}_{ij}+{\beta }_{2j}{(\mathrm{Arousal})}_{ij}+{r}_{ij}$$

Level-2:

2 $${\beta }_{0j}={\gamma }_{00}+ {\gamma }_{01}{\left(\mathrm{Chronotype}\right)}_{j}+{\gamma }_{02}{\left(\mathrm{Musical\,sophistication}\right)}_{j}+{u}_{0j}$$

3 $${\beta }_{1j}={\gamma }_{10}+ {\gamma }_{11}{(\mathrm{Chronotype})}_{j}$$

With the variance estimates *r* (level-1) and *u* (level-2). The same data post processing and model building steps were applied on the coefficient of variation of the ITIs, in order to check if the tapping variability was also influenced by the same factors. As the analysis of tapping variability was not the main aim of this study, results will only be mentioned briefly. The full documentation and results can be found in the supplementary materials.

## Results

### Mean SMT pace and variability

Across all participants and tests, the mean SMT was 650 ms (*SD* = 253 ms), ranging from 181 and 1822 ms. Figure 1 shows the distribution of the SMT for all responses and each time of the day relative to the first prompt aggregated over seven consecutive days. The overall mean tapping variability measured with the coefficient of variation was 9.25% (*SD* = 5.46).

### Multi-level analysis

**Fig. 2:**
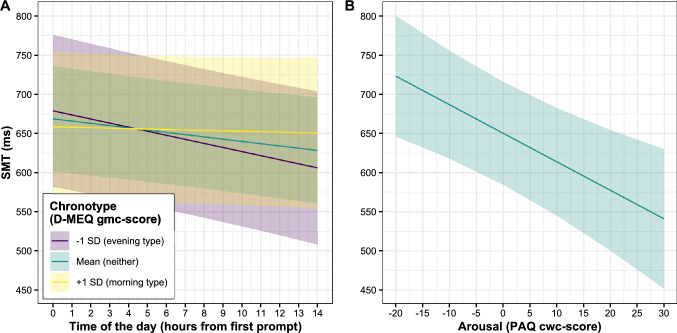
Main effects of time of the day (**A**), the interaction effect with chronotype (D-MEQ gmc-score; **A**), and arousal (PAQ cwc-score; **B**). The shaded areas represent 95% confidence intervals

In order to investigate the relationship between time of the day, chronotype (D-MEQ gmc-score), arousal (PAQ cwc-score), and musical sophistication (Gold-MSI gmc-score), a combined multi-level model was employed (Table 2). The ICC from the baseline model was 0.63, suggesting that 63% of variance stems from between participants and 37% from within participants. The final model resulted in a main effect of time of the day and shows that with each hour from the first prompt the SMT became on average 2.89 ms faster (Fig. 2A, green line). Thus, the SMT was slowest in the morning hours and became faster during the day. The interaction between time of the day and chronotype was also significant, suggesting that the pace acceleration of the SMT during the day depended on the chronotype. As Fig. 2A shows, the SMT of participants tending towards the morning type (+ 1 *SD* of D-MEQ gmc-score) stayed relatively constant and only slightly sped-up throughout the day (yellow line), whereas the SMT of participants tending towards the evening type (-1 *SD* of D-MEQ gmc-score) became faster (purple line). The main effect of chronotype was not significant, suggesting no general difference between chronotypes in the SMT. Arousal did result in a significant main effect, suggesting that with each point on the PAQ score the SMT became 3.65 ms faster. Thus, the more aroused the participants were, the faster was their SMT. Participants’ musical sophistication did not show a significant main effect on the SMT, suggesting no influence of participants’ musical experience on the SMT. As described in the model building process, all non-significant interactions were removed from the model; thus, none of the fixed factors further interacted with each other.

**Table 2:** Results of the multi-level analysis for the SMT

	Fixed					Random	
	Coeff	*β* [CI]	*SE*	*T*	*P*	Coeff	*SD*
**Intercept**	*γ*_00_	668.80 [599.71, 737.92]	34.40	19.44	> 0.001	*u*_0j_	199.36
Level-1							
**Time of the day**	*γ*_10_	− 2.89 [− 5.66, − 0.64]	1.14	− 2.53	0.012		
**Arousal**	*γ*_20_	− 3.65 [− 5.66, − 1.64]	1.03	− 3.56	> 0.001		
Level-2							
**Chronotype**	*γ*_01_	− 0.97 [− 7.74, 5.80]	3.37	− 0.29	0.774		
**Musical sophistication**	*γ*_02_	0.43 [− 2.49, 3.35]	1.45	0.30	0.770		
Cross-level							
Time of the day * Chronotype	*γ*_11_	0.22 [0.01, 0.44]	0.11	2.01	0.044		

R^2^_conditional_ = 63%, R^2^_marginal_ = 1%

Regarding the variability of the SMT, the final model resulted in no significant main effects of the fixed factors and no interaction (all *p* > 0.05). The best performing model was the unconditional one including a random slope for arousal. Furthermore, fixed factors did not significantly improve the model prediction, suggesting that the tapping variability of the SMT was not influenced by the time of the day, chronotype, arousal, and musical sophistication (see supplementary materials).

## Discussion

This study investigated the influence of time of the day, chronotype, and arousal on the pace of the spontaneous motor tempo (SMT). Using an experience sampling method with a finger-tapping paradigm, participants’ SMT was measured four times a day over seven consecutive days in their everyday life, by letting them tap at their most comfortable pace on the touchscreen of their smartphones. Furthermore, participants’ musical sophistication was assessed in order to control for potential differences. Results of a multi-level model suggest an influence of the time of the day such that the pace of the SMT became faster during the day. This effect interacted with the chronotype score, as participants tending towards a morning type (“larks”) were relatively faster in the morning and their SMT remained relatively constant during the day compared to more evening-type participants (“owls”), who were slower in the morning. Arousal affected the pace of the SMT as well. The more aroused participants were, the faster was the SMT. This effect did not interact with the other factors. These findings are in line with the chronobiological effects on the SMT and related tasks reported in previous studies (Hammerschmidt et al., 2021; Moussay et al., 2002; Tamm et al., 2009; Wright & Palmer, 2020), providing further support that the SMT may directly be influenced by the circadian rhythm of the biological clock, because fluctuations of the SMT over the day depend systematically on the combination of time of the day and participants’ 24-h sleep and activity cycle (i.e., chronotype).

The first hypothesis stated that the time of the day influences the pace of the SMT, which was confirmed by the results of the multi-level model. The SMT became on average 2.89 ms faster for every hour after the first prompt of the day, which was between 8 and 9 am, suggesting a pace acceleration during the day. Although potential confounding influences of factors affecting the time of the day effect such as sleep patterns as well as medication, drug, and caffeine consumption during the week cannot be ruled out entirely, these results confirm previous studies which also found a speeding-up of the SMT during the day (Hammerschmidt et al., 2021; Moussay et al., 2002). Moussay et al. (2002) also reported a slowing of the SMT between 6 and 10 pm, which had been expected due to the cyclic nature of the biological clock. Although a slight slowing in the pace of the SMT was observable in the current study, this slowing in the evening hours might have been less distinct compared to Moussay et al. due to the time period of data collection. Since the day length (number of sun hours) varies as a function of different seasons, data of these two studies might have been collected at different periods in the year. Data collection for this study was done in the summer with an average day length of about 16 h during the test week. These long sun hours might have mitigated the slowing of the SMT in the evening since it can affect sleep patterns. Another reason which might have caused this difference is the distribution of chronotypes. In this study, chronotypes were normally distributed (see supplementary materials), showing no skewness towards a particular chronotype, which might have been different in the sample by Moussay and colleagues and in turn affecting their results. Furthermore, a recent study on sleep patterns during the COVID-19 lockdown found a shift to later bedtimes and waking times (Gupta et al., 2020). Since the data collection for this study was carried out after the lockdown, this effect might have carried over to the post-lockdown period, resulting only in the small slowing of the SMT in the evening hours.

The second hypothesis was closely related to the first one and stated that the changes of the SMT during the day are also influenced by the chronotype, because previous research on chronotype-dependent performance provided evidence that different entrainments to the 24-h cycle, meaning the midpoint between sleep onsets, do indeed affect timing, sensorimotor, and cognitive capabilities (Tamm et al., 2009; van Vugt et al., 2013; Wright & Palmer, 2020). Thus, an interaction effect was expected between time of the day and chronotype. Results confirmed this hypothesis, since the D-MEQ score as a measure of chronotype showed a significant interaction with time of the day. Participants with a high D-MEQ score (morning type) showed a faster SMT in the morning hours compared to participants with a mid-range chronotype score (neither type) and participants with a low score (evening type). High D-MEQ score participants’ SMT became only slightly faster during the day, whereas participants’ SMT with a low score showed a much faster pace which indicates a shift in the phase reference. Thus, this study is the first to show that the inter-individual differences in the changes of the SMT during the day can partly be explained by the chronotype. Consequently, the SMT should be added to the list of sensorimotor chronotype-dependent performance tasks.

The significant interaction between time of the day and chronotype supports the assumption of a direct relationship between the pace of the SMT and the circadian rhythm and thus the biological clock. Furthermore, these results confirm previous studies reporting the same effect using different samples, paradigms (finger tapping, cycling), methods (lab-based and online), and test times (Hammerschmidt et al., 2021; Moussay et al., 2002). This shows a consistent influence of the time of the day on the pace of the SMT. Thus, results of this study suggest that the spontaneous and preferred rate of periodic motor actions is indeed influenced by the circadian rhythm of the biological clock.

The third hypothesis stated that higher arousal leads to a faster SMT pace. This was confirmed by a main effect of arousal. The results show that a higher PAQ score (Dieleman et al., 2010) resulted in a faster SMT with a 3.65-ms increase with each PAQ score point. This is in line with previous studies suggesting the same effect for induced arousal using auditory stimuli (Boltz, 1994; Perilli, 1995) and self-rated felt arousal (Hammerschmidt et al., 2021). It supports the finding of a faster SMT after physical exercise (i.e., increased physiological arousal) compared to before (Dosseville et al., 2002), and a faster prefered perceptual tempo (Jakubowski et al., 2015), in contrast to a study which found no such differences (Sysoeva et al., 2013). Since the PAQ measures self-perceived physiological states (e.g., sweatiness, moisture of the mouth), it can be assumed that physiological changes in the body (e.g., heart rate, cortical blood flow) speed up the SMT. It further supports the *Sympathetic Hypothesis,* stating that higher physiological arousal increases the preferred perceived tempo and thus the SMT as well (Holbrook & Anand, 1990).

Musical sophistication, measured with the general factor of *Musical Sophistication Index* (Müllensiefen et al., 2014), did not show an effect on the pace of the SMT. Previous studies reported differences in the SMT based on musical experience. One study reported a slower SMT for children with musical training compared to children with no musical training, yet this effect could not be found in adults (Drake et al., 2000). Results of an online study did find that musically more experienced participants regarding playing a musical instrument preferred a slower SMT. Yet, when grouped into six different SMT clusters ranging from a very fast to very slow pace, the same study found the opposite effect, as a slow cluster showed the least musical experience (Hammerschmidt et al., 2021). In the current study, a more complex measure for musical sophistication was used showing no effect on the SMT. On the one hand, these seemingly contrasting results may partly be caused by the different measures used in the studies. The general factor of the Gold-MSI is mostly associated with the sub-scales Musical Training and Singing Ability which are closely related to the measures used in other studies. Thus, it cannot be ruled out that other ways which are characterized as musical and are not represented in these measures such as timing skills affect the SMT (Baker et al., 2020). On the other hand, it seems more likely that musical experience does not affect the SMT given the lack of explanation for how enhanced cognitive abilities from musical training should transfer to the relatively simple motor task of tapping to the SMT. In line with this, a study did find differences based on musical experience in the spontaneous production rate (SPR) when tapping the rhythms of melodies (Scheurich et al., 2018), whereas no such difference in the SPR could be found in another recent study when participants tapped isochronously rather than rhythmically (Scheurich et al., 2020). This also suggests that the influence of musical training depends on the complexity of the tapping task. For example, SMT is different from sensorimotor synchronization (SMS) for which clear effects of musical training occur, especially in more complex SMS tasks (e.g., Hammerschmidt & Wöllner, 2020; Repp, 2010). SMS involves prediction, error correction, and adaptive timing (van der Steen & Keller, 2013), processes which do not directly affect the rather lower-level SMT task. Furthermore, this is supported since the variability of participants’ tapping was not influenced by any of the factors. Because the coefficient of variation was used for the analysis, thus controlling for different paces of the SMT, it can be assumed that only the pace of the SMT affects its variability. Tapping variability typically increases with slower tempos in sensorimotor synchronization tasks (Repp, 2005; Repp & Doggett, 2007). In contrast, SMT is unique in that musical sophistication did not influence the variability at the most comfortable tapping rate, which is usually the case in sensorimotor synchronization tasks.

The combination of an experience sampling method and the self-developed web application has proven to be a valuable tool for the assessment of the SMT in everyday-life contexts and offers further solutions for the usage of finger-tapping paradigms in out-of-the-lab scenarios. On the other hand, limitations of experience sampling method and online studies lie undoubtedly in the observability and control for task comprehension and execution. In this study, these circumstances were accounted for by a detailed introduction event. Furthermore, the different technical devices might have had an influence on response collection, yet this influence should be of little concern as internally varying latencies in one and the same device were not of interest, because synchronization accuracy was not measured, for which latencies would need to be controlled. In line with this, a previous study using the same tapping application did not find differences between different hard- and software types on the pace of the SMT using a large sample (Hammerschmidt et al., 2021). However, future studies may use the same device in order to reach more control of the response collection process, particularly if synchronization is also analyzed. Future studies could also collect data on sleep patterns and disorders, as well as medication, drug and caffeine usage, in order to reach further control of potential factors affecting the circadian rhythm.

To conclude, this study investigated chronobiological effects on the spontaneous motor tempo (SMT) in participants’ everyday life with an experience sampling method. Results show that the SMT varied according to the time of the day. Furthermore, this study is the first to show that these changes in the SMT depend on someone’s chronotype, as a gradual phase shift in the chronotype score between “larks” (morning types) and “owls” (evening types) was observed. Thus, these results indicate an influence of the circadian rhythm on the SMT, which in turn suggests that the SMT is an estimate of the biological clock. Furthermore, self-assessed physiological arousal in the body caused the SMT to speed-up, an effect which was independent from the time of the day and chronotype in this study. Given the assumption of a shared mechanism for perceptual and rhythmic motor behaviors (Kliger Amrani & Zion Golumbic, 2020; McAuley et al., 2006), these results suggest that tempo preferences and temporal acuity when performing music may also be influenced by the biological clock (cf., Wright & Palmer, 2020); thus, there might be an ideal time to practice a certain piece of music based on its tempo. Regarding auditory perception, tempo preference when listening to music may change as a function of the time of day, and thus it should be a useful criterion for background music choice in public, in order to align the musical tempo with listeners’ biological clock. 

Beyond a musical context, the SMT generally indicates the “sweet spot” of temporal predictability necessary for the processing of upcoming auditory stimuli such as language, since temporal acuity is related to attention and working memory (Ding et al., 2017; Jones & Boltz, 1989; Kliger Amrani & Zion Golumbic, 2020). Thus, the optimal timing regarding the temporal aspects of these cognitive processes may change as a function of someone’s biological clock, an assumption which needs further testing. Therefore, the outcomes of this study are informative for the psychology of time, chronobiology, as well as for music and language perception and production.

## Supplementary Information

Below is the link to the electronic supplementary material.

Supplementary file1 (DOCX 171 KB)

## Data Availability

The data supporting the conclusions of this article are openly avaible in Zenodo repository at 10.5281/zenodo.5947002.
