# Supplementary material for: Spontaneous motor tempo over the course of a week: the role of the time of the day, chronotype, and arousal
Source: Psychol Res. 2022 Feb 6;87(1):327–38. doi: 10.1007/s00426-022-01646-2 (PMC8818276; doi:10.1007/s00426-022-01646-2)
Supplement: Supplementary file 1 — Supplementary file1 (DOCX 171 KB) [file 426_2022_1646_MOESM1_ESM.docx]

Supplementary Materials –

Spontaneous motor tempo over the course of a week: The role of the time of the day, chronotype, and arousal

David Hammerschmidt^1^, Clemens Wöllner^1^

^1^Institute for Systematic Musicology, University of Hamburg, Hamburg, Germany

Corresponding author:

David Hammerschmidt, Institute for Systematic Musicology, University of Hamburg, Alsterterrasse 1, 20354 Hamburg, Germany

Email: [david.hammerschmidt@uni-hamburg.de](mailto:david.hammerschmidt@uni-hamburg.de)

Supplementary Table 1. Distribution of participants’ German school degrees.

| **School degree** | **N** | **Percentage** |
| --- | --- | --- |
| Hauptschule (lower secondary high school) | 1 | 2.8 |
| Realschule (secondary high school) | 2 | 13.9 |
| Fachoberschule (higher secondary vocational school) | 6 | 16.7 |
| Gymnasium (academic high school) | 24 | 66.6 |

Supplementary Table 2. Distribution of participants’ higher education.

| **Higher education** | **N** | **Percentage** |
| --- | --- | --- |
| No apprenticeship / university degree | 1 | 2.8 |
| Currently an apprentice / trainee / university student | 8 | 22.2 |
| Apprenticeship (firm / business) | 5 | 13.9 |
| Apprenticeship (vocational school) | 1 | 2.8 |
| Master apprenticeship training | 1 | 2.8 |
| College degree (university of applied science) | 3 | 8.3 |
| Bachelor degree | 10 | 27.8 |
| Master degree | 7 | 19.4 |

Supplementary Table 3. Distribution of participants’ area of profession.

| **Profession** | **N** | **Percentage** |
| --- | --- | --- |
| Transport / logistics | 1 | 2.8 |
| Production / fabrication | 1 | 2.8 |
| Construction / architecture / survey | 1 | 2.8 |
| IT / computer | 2 | 5.5 |
| Economics / administration | 2 | 5.5 |
| Media | 3 | 8.3 |
| Art / culture / design | 5 | 13.9 |
| Service | 6 | 16.7 |
| Science / academia | 6 | 16.7 |
| Social / education | 9 | 25.0 |

Supplementary Table 4 Distribution of participants’ employment status.

| **Employment status** | **N** | **Percentage** |
| --- | --- | --- |
| Full-time employed | 14 | 38.9 |
| Marginal employed (mini job) | 6 | 16.7 |
| Part-time employed | 6 | 16.7 |
| Self-employed | 6 | 16.7 |
| Not employed | 4 | 11.0 |


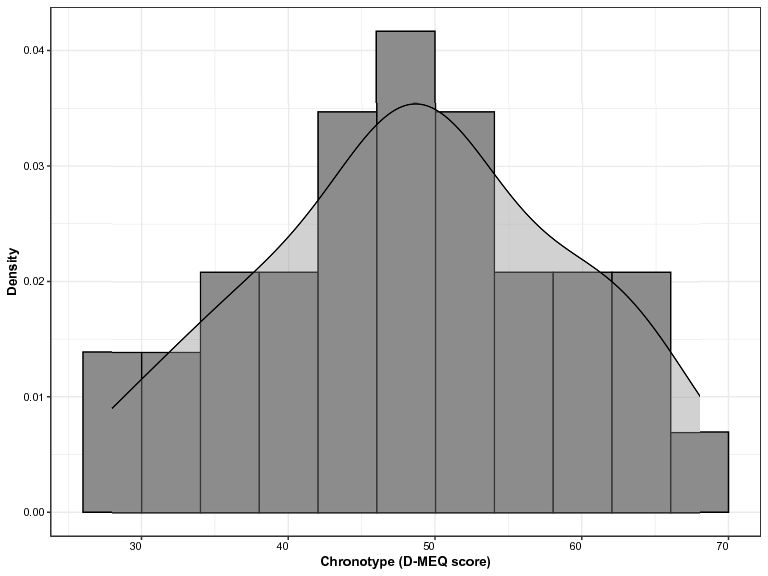


Supplementary Figure 1. Distribution of participants‘ D-MEQ score indicating the chronotype scores; scores 16–41 = evening type, scores 42–58 = neither type, and scores 59–86 = morning type. Bar width represents 4 score points on the scale.


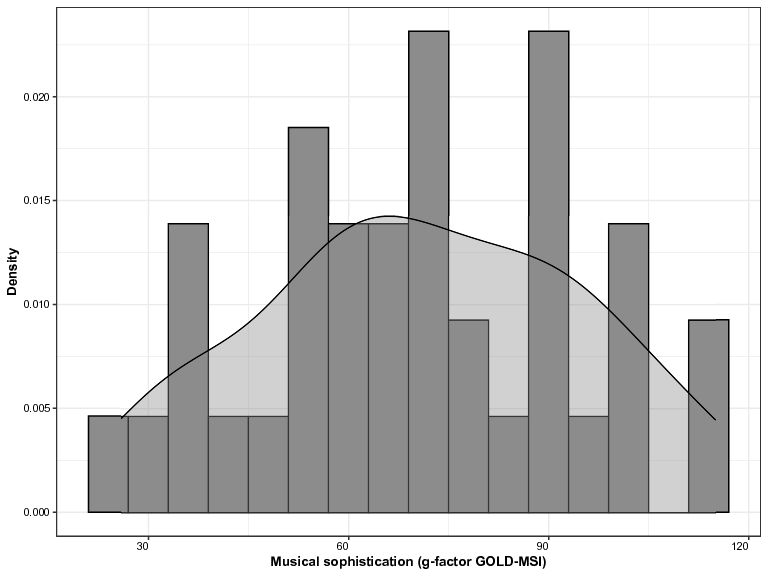


Supplementary Figure 2. Distribution of participants‘ g-factor score of the GOLD-MSI indicating musical sophistication. Bar width represents 6 score points on the scale.


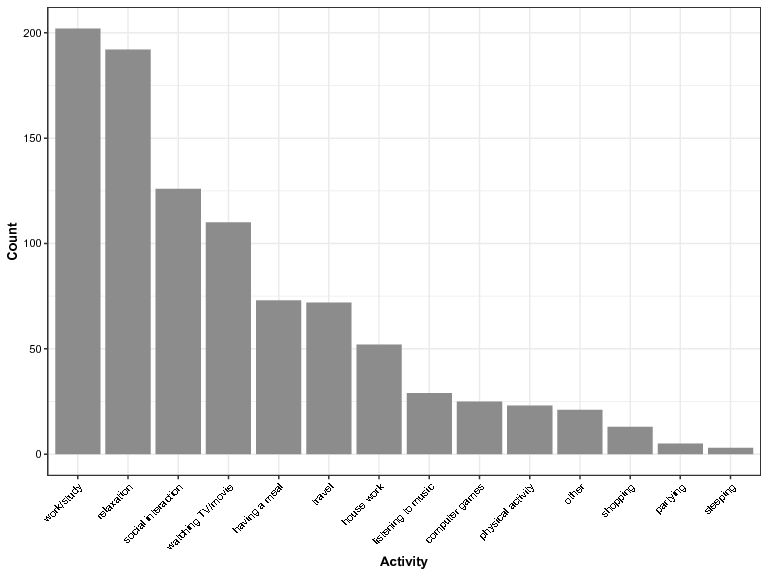


Supplementary Figure 3. Distributions of participants’ activities during the tests.


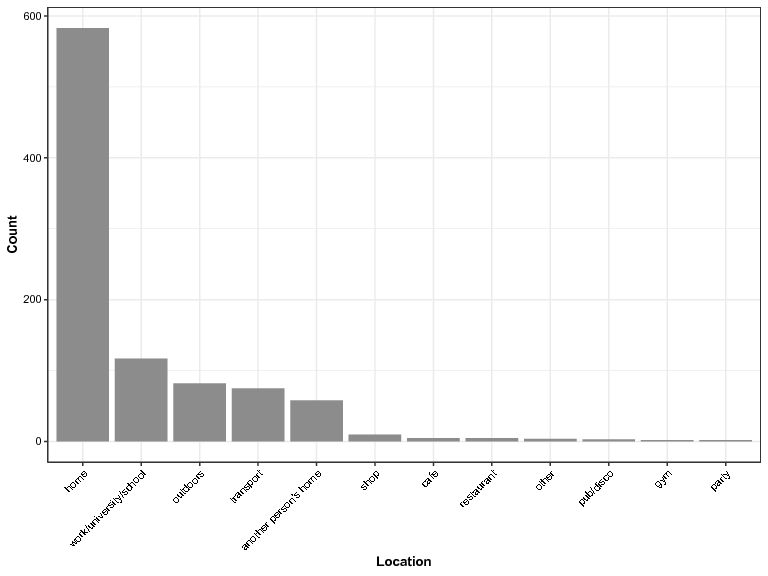


Supplementary Figure 4 Distributions of participants’ locations during the tests.


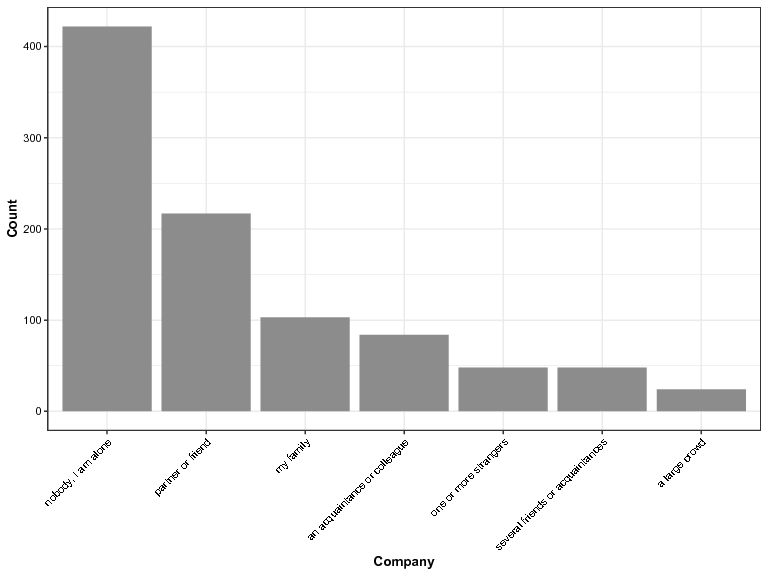


Supplementary Figure 5. Distributions of participants’ company during the tests.

**Model building: Tapping variability (coefficient of variation)**

Before a multilevel model analysis was carried out on the coefficient of variation, an outlier detection per participant was applied using 1.5 IQR. This resulted in further exclusion of *N* = 65 trials. All level-1 variables were again entered first as random factors. The random effects covariance matrices resulted in singular fits (i.e., random variance close to zero) for time of the day, and hence this variable was not further included as a random factor. The intraclass correlation was 36%.

Supplementary Table 5. Performance measures for the multi-level model building.

| Models | Number of parameters | AIC | BIC | Log Likelihood | Deviance | *p* |
| --- | --- | --- | --- | --- | --- | --- |
| Unconditional | 3 | 5197 | 5212 | -2596 | 5191 |  |
| *Unconditional + random slope | 5 | 5180 | 5204 | -2585 | 5170 | < .001 |
| Fixed factors | 9 | 5184 | 5227 | -2583 | 5166 | .427 |
| Fixed factors + all interactions | 15 | 5193 | 5265 | -2582 | 5163 | .768 |

Note. Model comparisons (p-values) were done sequentially to the one below. The asterisk indicates the best performing and final model.

Supplementary Table 6. Results of the multi-level analysis of the Fixed factors model for the coefficient of variation.

|  | Fixed |  |  |  |  | Random |  |
| --- | --- | --- | --- | --- | --- | --- | --- |
|  | Coeff. | *b* (CI) | *SE* | *t* | *p* | Coeff. | *SD* |
| Intercept | *γ*_00_ | 8.91 (7.73, 10.09) | 0.59 | 15.11 | < .001 | *u*_0j_ | 3.19 |
| Level-1 |  |  |  |  |  |  |  |
| Time of the day | *γ* _10_ | 0.04 (-0.02, 0.01 | 0.03 | 1.23 | .22 |  |  |
| Arousal | *γ* _20_ | 0.03 (-0.06, 0.12) | 0.04 | 0.71 | .48 | *u*_1j_ | 0.17 |
| Level-2 |  |  |  |  |  |  |  |
| Chronotype | *γ* _01_ | -0.02 (-0.13, 0.09) | 0.05 | -0.36 | .72 |  |  |
| Musical sophistication | *γ* _02_ | -0.03 (-0.08, 0.02) | 0.02 | -1.41 | .17 |  |  |
